# Transcranial Laser Therapy Does Not Improve Cognitive and Post-Traumatic Stress Disorder–Related Behavioral Traits in Rats Exposed to Repetitive Low-Level Blast Injury

**DOI:** 10.1089/neur.2021.0005

**Published:** 2021-12-02

**Authors:** Georgina Perez Garcia, Gissel M. Perez, Alena Otero-Pagan, Rania Abutarboush, Usmah Kawoos, Rita De Gasperi, Miguel A. Gama Sosa, Dylan Pryor, Patrick R. Hof, David G. Cook, Sam Gandy, Stephen T. Ahlers, Gregory A. Elder

**Affiliations:** ^1^ Research and Development Service, James J. Peters Department of Veterans Affairs Medical Center, Bronx, New York, USA.; ^2^ Department of Neurology, Icahn School of Medicine at Mount Sinai, New York, New York, USA.; ^3^ Department of Neurotrauma, Naval Medical Research Center, Silver Spring, Maryland, USA.; ^4^ The Henry M. Jackson Foundation for the Advancement of Military Medicine, Inc., Bethesda, Maryland, USA.; ^5^ Department of Psychiatry, Icahn School of Medicine at Mount Sinai, New York, New York, USA.; ^6^ General Medical Research Service, James J. Peters Department of Veterans Affairs Medical Center, Bronx, New York, USA.; ^7^ Nash Family Department of Neuroscience and Friedman Brain Institute, Icahn School of Medicine at Mount Sinai, New York, New York, USA.; ^8^ Department of Geriatrics and Palliative Care, Icahn School of Medicine at Mount Sinai, New York, New York, USA.; ^9^ Mount Sinai Alzheimer's Disease Research Center and Ronald M. Loeb Center for Alzheimer's Disease, Icahn School of Medicine at Mount Sinai, New York, New York, USA.; ^10^ Geriatric Research Education and Clinical Center, VA Puget Sound Health Care System, Seattle, Washington, USA.; ^11^ Department of Medicine, University of Washington, Seattle, Washington, USA.; ^12^ Barbara and Maurice A. Deane Center for Wellness and Cognitive Health and the Mount Sinai NFL Neurological Care Center, Icahn School of Medicine at Mount Sinai, New York, New York, USA.; ^13^ Neurology Service, James J. Peters Department of Veterans Affairs Medical Center, Bronx, New York, USA.

**Keywords:** blast, post-traumatic stress disorder, rat, transcranial laser therapy, traumatic brain injury

## Abstract

Many military veterans who experienced blast-related traumatic brain injuries (TBIs) in the conflicts in Iraq and Afghanistan suffer from chronic cognitive and mental health problems, including post-traumatic stress disorder (PTSD). Transcranial laser therapy (TLT) uses low-power lasers emitting light in the far- to near-infrared ranges. Beneficial effects of TLT have been reported in neurological and mental-health–related disorders in humans and animal models, including TBI. Rats exposed to repetitive low-level blast develop chronic cognitive and PTSD-related behavioral traits. We tested whether TLT treatment could reverse these traits. Rats received a 74.5-kPa blast or sham exposures delivered one per day for 3 consecutive days. Beginning at 34 weeks after blast exposure, the following groups of rats were treated with active or sham TLT: 1) Sham-exposed rats (*n* = 12) were treated with sham TLT; 2) blast-exposed rats (*n* = 13) were treated with sham TLT; and 3) blast-exposed rats (*n* = 14) were treated with active TLT. Rats received 5 min of TLT five times per week for 6 weeks (wavelength, 808 nm; power of irradiance, 240 mW). At the end of treatment, rats were tested in tasks found previously to be most informative (novel object recognition, novel object localization, contextual/cued fear conditioning, elevated zero maze, and light/dark emergence). TLT did not improve blast-related effects in any of these tests, and blast-exposed rats were worse after TLT in some anxiety-related measures. Based on these findings, TLT does not appear to be a promising treatment for the chronic cognitive and mental health problems that follow blast injury.

## Introduction

Traumatic brain injury (TBI) is common in civilian and military life. Public awareness of military-related TBI increased recently because of the conflicts in Iraq and Afghanistan.^1^ Military-related TBIs occur through various mechanisms. However, certain types of TBI are relatively unique to the military, the most prominent being TBI related to blast injury. Indeed, exposures to mortars, artillery shells, and improvised explosive devices constituted the major cause of TBI in Iraq and Afghanistan.^1–3^ Potential consequences of blast-related TBI include neurological symptoms that may evolve into a chronic post-concussion syndrome that can persist for years.^1^ Besides static symptoms, new symptoms may develop or existing ones may worsen.^4,5^ Blast-related TBI may also be a risk factor for the later development of neurodegenerative diseases.^6–9^ In addition to the overt exposures associated with clinically recognized TBI, there are also concerns over potential adverse effects of what is now being referred to as military occupational blast exposure, a type of subclinical blast exposure, that is common for many service members in combat as well as non-combat settings.^10–12^

Transcranial laser therapy (TLT) uses low-power lasers and light-emitting diodes (LEDs) in the far- to near-infrared domain of the light spectrum.^13^ The low-level lasers and LEDs used do not emit significant heat, but modulate numerous cellular activities, including increasing mitochondrial function, enhancing adenosine triphosphate synthesis, altering intracellular calcium, and modulating reactive oxygen species production.^14–16^ Beneficial effects of TLT have been shown in a range of neurological disorders in humans and animal models, including TBI, stroke, Alzheimer's disease, and Parkinson's disease.^13,17–20^ There is also hope that TLT will be effective in the treatment of major depression,^18^ and some evidence suggests that TLT may even enhance cognitive performance in healthy controls.^13^

Rats exposed to repetitive low-level blast develop cognitive and post-traumatic stress disorder (PTSD)-related behavioral traits that are present for at least 1 year after exposure.^21–25^ Traits develop in a delayed manner, being absent in the first 8 weeks after blast exposure but present 12 weeks and longer after exposure.^25^ These rats model the chronic blast-associated neurocognitive and behavioral syndromes that occur in military veterans.^1^

Given the reported beneficial effects of TLT in treatment of non-blast-related TBI in humans and animals^17,26–32^ as well as one report in a blast injury model,^33^ we tested whether TLT treatment could reverse cognitive and PTSD-related behavioral traits that develop after repetitive low-level blast exposure. We found that a 6-week course of TLT did not improve these traits.

## Methods

### Animal assurance

The study protocol was reviewed and approved by the institutional animal care and use committees of the Walter Reed Army Institute of Research (WRAIR)/Naval Medical Research Center (Silver Spring, MD) and the James J. Peters VA Medical Center (Bronx, NY). Experiments were conducted in compliance with the Animal Welfare Act and per the principles set forth in the Guide for Care and Use of Laboratory Animals (Institute of Laboratory Animals Resources, National Research Council, National Academy Press, 2011).

### Animals

Adult male Long-Evans hooded rats (250–350 g; 10 weeks of age; Charles River Laboratories International, Wilmington, MA) were used.

### Blast overpressure exposure

Rats were exposed to overpressure injury using a shock tube, which simulates the effects of air blast exposure under experimental conditions. The shock tube has a 0.32-m circular diameter and is a 5.94-m-long steel tube divided into a 0.76-m compression chamber separated from a 5.18-m expansion chamber. The compression and expansion chambers are separated by polyethylene terephthalate Mylar^TM^ sheets (DuPont, Wilmington, DE) that control the peak pressure generated. Peak pressure at the end of the expansion chamber was determined with piezoresistive gauges specifically designed for pressure-time (impulse) measurements (Model 102M152; PCB Piezotronics, Depew, NY).

Individual rats were anesthetized using an isoflurane gas anesthesia system consisting of a vaporizer, gas lines, and valves and an activated charcoal-scavenging system adapted for use with rodents. Rats were placed into a polycarbonate induction chamber, which was closed and immediately flushed with 5% isoflurane mixture in air for 2 min. Rats were placed into a cone-shaped plastic restraint device and then placed in the shock tube. Movement was further restricted during the blast exposure using 1.5-cm-diameter flattened rubber tourniquet tubing. Three tourniquets were spaced evenly to secure the head region, the upper torso, and lower torso while the animal was in the plastic restraint cone. The end of each tubing was threaded through a toggle and run outside of the exposure cage where it was tied to firmly affix the animal and prevent movement during the blast overpressure (BOP) exposure without restricting breathing. Rats were randomly assigned to sham or blast conditions and were placed in the shock tube lying prone with the plane representing a line from the tail to the nose of the body in line with the longitudinal axis of the shock tube with the head placed more upstream. Total length of time under anesthesia, including placement in the shock tube and execution of the blast procedure, was typically <3 min.

Blast-exposed animals received 74.5 kPa (equivalent to 10.8 psi, duration 4.8 ms, and impulse 175.8 kPa*ms) exposures administered one exposure per day for 3 consecutive days. Further details of the physical characteristics of the blast wave are described in Ahlers and colleagues.^34^ Sham (control) exposed rats were treated identically, including receiving anesthesia and being placed in the blast tube, but did not receive a blast exposure. Three days after the last blast or sham exposure, animals were transported in a climate-controlled van from the WRAIR to the James J. Peters VA Medical Center. Animals left in the morning from the WRAIR and arrived in the afternoon of the same day at the James J. Peters VA Medical Center, where all other procedures were performed.

### Animal housing

Animals were housed at a constant 70–72^o^F temperature with rooms on a 12:12-h light cycle with lights on at 7:00 am. All subjects were individually housed in standard clear plastic cages equipped with Bed-O'Cobs laboratory animal bedding (The Andersons, Maumee, OH) and EnviroDri nesting paper (Sheppard Specialty Papers, Milford, NJ). Access to food and water was *ad libitum*. Subjects were housed on racks in random order to prevent rack position effects. Cages were coded to allow maintenance of blinding to groups during behavioral testing.

### Transcranial laser therapy

TLT was based on published protocols in rats.^35,36^ The laser device was a gallium aluminum arsenide (GaAIAs) diode laser with a compact controller for pigtail lasers (CLD1010LP; Thorlabs Inc., Newton, NJ). A function/waveform generator (33500B; Keysight, Santa Rosa, CA) was connected to the GaAIAs diode and an SM Fiber Pigtail (LP820-SF80; Thorlabs) connected to a Fiber Collimation device (F280FC-850; Thorlabs) was used to deliver radiation to the head surface.

**FIG. 1.**
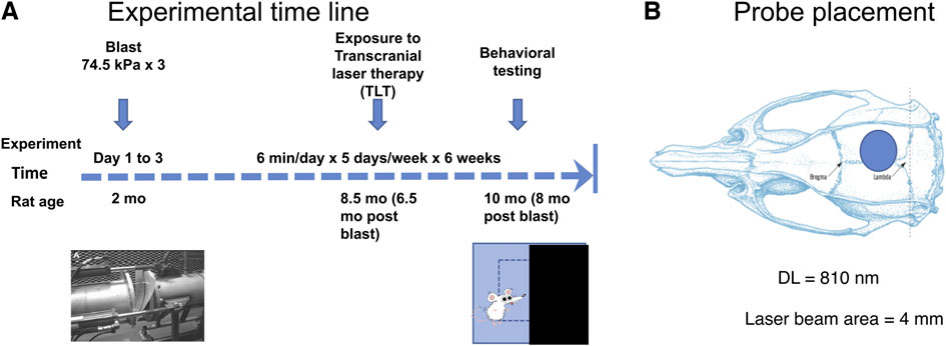
Transcranial laser therapy (TLT) of blast- and sham-exposed rats. (**A**) Design and timeline of experiments. At 2 months of age, rats were exposed to three 74.5-kPa blast or sham exposures delivered one per day for 3 consecutive days. Beginning 6.5 months after blast or sham exposure, rats received active or sham TLT for 6 min/d, delivered 5 days per week for 6 weeks. After treatment, rats were tested in light/dark escape, elevated zero maze, novel object recognition, novel object localization, and fear conditioning tasks. (**B**) Probe laser beam placement, which radiated over a 4-mm area between bregma and lambda. DL, dorsolateral.

Rats were habituated to being restrained in 5-min sessions daily for 1 week before beginning TLT at week 34 post-blast. The laser probe/collimator was held 0.05 mm over the head in the midline of the dorsal surface of the rat head midway between bregma and lambda (Fig. 1). The laser was operated in a pulsed wave (PW) mode on 10 Hz, with an 88% duty cycle. Rats were exposed to an 808-nm wavelength, 10-Hz PW with an amplitude of 2.35 Vrms, delivered to a 4-mm circular area for 5 min/d, 5 days per week for 6 weeks. Average irradiance was 240 mW. Laser wavelength and signal intensity were confirmed with VCR4 detector cards (Thorlabs). For sham TLT, rats were restrained in the same way for an equal amount of time and the diode/collimator was placed over the head, but the laser was not turned on.

### Elevated zero maze

The elevated zero maze (EZM) apparatus consisted of a circular black Plexiglas runway 121.92 cm in diameter and raised 76 cm off the floor (San Diego Instruments, San Diego, CA). The textured runway itself was 5.08 cm across and divided equally into alternating quadrants of open runway enclosed only by a 1.27-cm lip and closed runway with smooth 15.24-cm walls. All subjects received a 5-min trial beginning in a closed arc of the runway. During each trial, subjects were allowed to move freely around the runway, with all movement tracked automatically by a video camera placed on the ceiling directly above the maze. Data were analyzed by ANYMAZE (San Diego Instruments), yielding measures of total movement time and distance for the entire maze, as well as time spent and distance traveled in each of the individual quadrants. From the quadrant data, measures of total open and closed arc times, latency to enter an open arm, total open arm entries, and latency to completely cross an open arc between two closed arcs were calculated. Subject position was determined by centroid location. Testing was conducted on 2 consecutive days.

### Light/dark emergence

A light/dark emergence (LD) task was run in Versamax activity cages with opaque black Plexiglas boxes enclosing the left half of the interiors so that only the right sides were illuminated. Animals began in the dark side and were allowed to freely explore for 10 min with access to the left (light) side through an open doorway located in the center of the monitor. Subject side preference and emergence latencies were tracked by centroid location with all movement automatically tracked and quantified. Light-side emergence latency, time to reach the center of the lighted side (light-side center latency), and percent total light-side duration were calculated from beam breaks. All equipment was wiped clean between tests.

### Novel object recognition

Rats were habituated to the arena (90 cm length × 60 cm width × 40 cm height) for 20 min, 24 h before training. On the training day, two identical objects were placed on opposite ends of the empty arena, and the rat was allowed to explore the objects freely for 7 min. After a 1-h delay, during which the rat was held in its home cage, one of the two familiar objects was replaced with a novel one, and the rat was allowed to freely explore the familiar and novel object for 5 min to assess short-term memory (STM). After a 24-h delay, during which the rat was held in its home cage, one of the two familiar objects was replaced with a novel one different from the ones used during the STM. The rat was allowed to freely explore the familiar and novel object for 5 min to assess long-term memory (LTM). Raw exploration times for each object are expressed in seconds. Object exploration was defined as sniffing or touching the object with the vibrissae or when the animal's head was oriented toward the object with the nose placed at a distance of <2 cm from the object.

All sessions were recorded by video camera (Sentech, Carrollton, TX) and analyzed with ANYMAZE software (San Diego Instruments). In addition, offline analysis by an investigator blind to the blast-exposed status of animals was performed. Objects to be discriminated were of different size, shape, and color and were made of plastic or metal material. Objects consisted of a 330-mL soda can, metal box, cup, and plastic tube. All objects were cleaned with 70% ethanol between trials.

### Contextual and cued fear conditioning

Sound-attenuated isolation cubicles (Coulbourn Instruments, Holliston, MA) were utilized. Each cubicle was equipped with a grid floor for delivery of the unconditioned stimulus (US) and overhead cameras. All aspects of the test were controlled and monitored by the Freeze Frame conditioning and video tracking system (Actimetrics; Coulbourn Instruments). During training, chambers were scented with almond extract, lined with white paper towels, had background noise generated by a small fan, and were cleaned before and between trials with 70% ethanol. Each subject was placed inside the conditioning chamber for 2 min before the onset of a conditioned stimulus (CS; an 80-dB, 2-kHz tone), which lasted for 20 sec with a coterminating 2-sec footshock (0.7 mA; US). A total of three tone/shock pairings were administered with the first/second and second/third separated by 1 min. Each rat remained in the chamber for an additional 40 sec after the third CS-US pairing before being returned to its home cage. Freezing was defined as a lack of movement (except for respiration) in each 10-sec interval. Minutes 0–2 during the training session were used to measure baseline freezing.

Contextual fear memory testing was performed 24 h after the training session by measuring freezing behavior during a 4-min test in the conditioning chamber under conditions identical to those of the training session with the exception that no footshock or tone (CS or US) was presented. Animals were returned to their home cage for another 24 h, at which time cued conditioning was tested. To create a new context with different properties, chambers were free of background noise (fan turned off), lined with blue paper towels, scented with lemon extract, and cleaned before and during all trials with isopropanol. Each subject was placed in this novel context, and baseline freezing was measured for 2 min, followed by exposure to the CS (20-sec tone) at 120 and 290 sec.

### Tissue processing and immunohistochemistry

Animals were euthanized after behavioral testing was completed. After deep anesthesia with a solution of 200 mg/kg of ketamine and 30 mg/kg of xylazine, rats were euthanized by transcardial perfusion with cold 4% paraformaldehyde in phosphate-buffered saline (PBS). After perfusion, brains were removed and post-fixed in 4% paraformaldehyde for 48 h, transferred to PBS, and stored at 4°C until sectioning. Forty-micrometer-thick coronal sections were cut through the entire extent of the hippocampus using a Leica VT1000 S Vibratome (Leica Microsystems, Wetzlar, Germany). Sections were stored at −20°C in a cryoprotectant solution (25% ethylene glycol and 25% glycerin [in 0.05 M of PBS]) until processing for immunofluorescence.

For collagen IV and ionized calcium-binding adaptor molecule 1 (Iba1) staining, sections from each brain were washed 4 × 5 min with PBS, then were incubated in blocking buffer (3% goat serum, 0.3% Triton X-100 in PBS) for 1 h and incubated overnight at 4°C in a mixture of rat anti–collagen IV rabbit (1:300; Abcam, Cambridge, MA) plus Iba1 (1:500; Wako Chemicals USA, Richmond, VA) antibodies. The next day, sections were washed 4 × 5 min with PBS and exposed for 2 h in the dark with Alexa Fluor 568–conjugated donkey antirat immunoglobulin G (IgG) and with Alexa Fluor 488–conjugated goat antirabbit IgG (Life Technologies, Carlsbad, CA). Both secondary antibodies were used at a dilution of 1:300. To ascertain the effects of TLT on phospho-tau accumulation and microglial accumulation, a second series of sections from each animal was immunolabeled with phospho-tau and glial fibrillary acidic protein (GFAP), as described above, using anti-AT270 antibody (1:500; ThermoFisherScientific, Waltham, MA) and chicken anti-GFAP (1:500; Novus Biologicals, Littleton, CO). The following day, secondary antibodies were applied as described above (Alexa Fluor antichicken and Alexa Fluor antirabbit; both from Life Technologies). All sections were mounted onto slides and covered under Fluoro-Gel with Tris Buffer (Electron Microscopy Sciences, Hatfield, PA). Images were acquired using a confocal microscope (Carl Zeiss AG, Jena, Germany).

### Statistical analysis

Values are expressed as mean ± standard error of the mean. Comparisons were performed using one-way analysis of variance (ANOVA), repeated-measures ANOVA, or unpaired *t*-tests. For *post hoc* tests after a significant one-way ANOVA, Fisher's least significant difference (LSD) was used. When repeated-measures ANOVA was used, sphericity was assessed using the Mauchly test. If the assumption of sphericity was violated (*p* < 0.05), significance was determined using the Greenhouse-Geisser correction. Statistical tests were performed using the program, GraphPad Prism (version 8.0; GraphPad Software Inc., La Jolla, CA), or SPSS software (v27; IBM Corp., Armonk, NY).

## Results

### Experimental design for blast exposure and transcranial laser therapy treatment

Figure 1 shows the experimental design and timeline of the experiment. Ten-week adult male Long-Evans rats received three 74.5-kPa exposures delivered once per day on 3 consecutive days. Non-blast-exposed controls were treated identically, including receiving anesthesia and being placed in the blast tube, but did not receive a blast exposure. Beginning at 34 weeks after blast exposure, the following groups of rats were treated with active or sham TLT: 1) Sham exposed rats (*n* = 12) were treated with sham TLT; 2) blast-exposed rats (*n* = 13) were treated with sham TLT; and 3) blast-exposed rats (*n* = 14) were treated with active TLT. Comparison of sham blast + sham TLT-treated controls with blast + sham TLT animals provided a positive control for development of the blast-related behavioral phenotype. Comparing blast + sham TLT with blast + active TLT-treated animals allowed effects of TLT on the blast-related phenotype to be determined. At the end of TLT treatment, rats were tested in tasks found previously to be most informative (novel object recognition [NOR], novel object localization [NOL], contextual/cued fear conditioning, EZM, and LD).^21–25^

### Transcranial laser therapy does not improve recognition or spatial memory deficits in blast-exposed rats

**FIG. 2.**
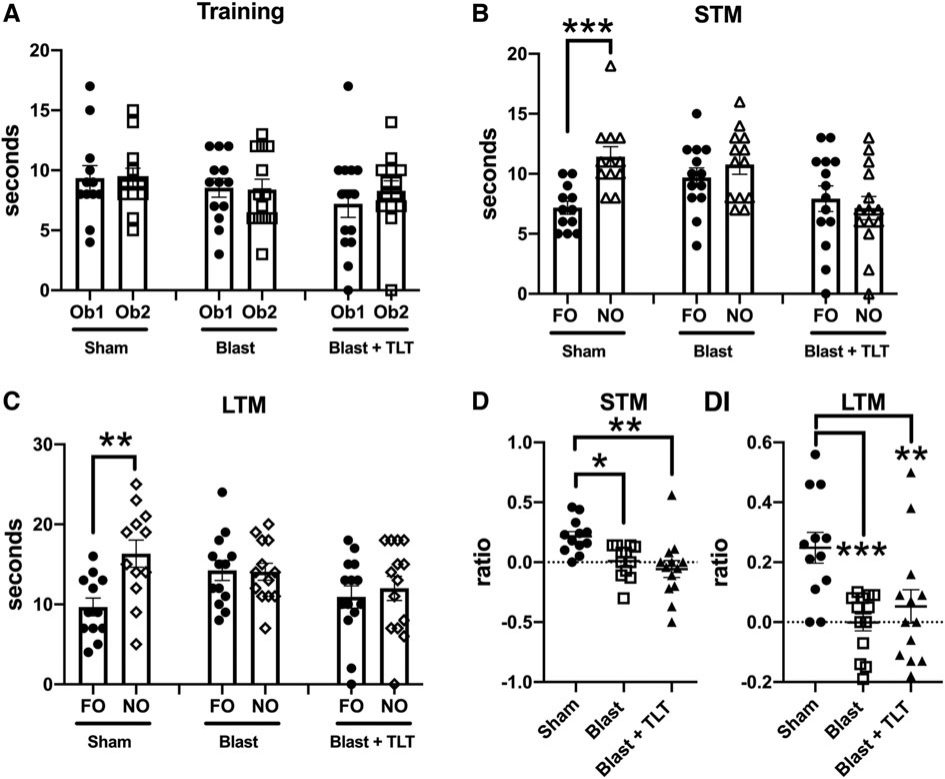
Novel object recognition (NOR) testing of blast-exposed rats treated with TLT. Sham and blast-exposed rats were treated with sham TLT (sham and blast; *n* = 12 sham, *n* = 13 blast) or active TLT (blast + TLT; *n* = 14). Rats were tested in NOR at the end of treatment. (**A**) Time spent exploring the objects (Ob1 and Ob2) during the training session. (**B,C**) Exploration of the previously presented familiar object (FO) compared to the novel object (NO) when presented 1 h (A, short-term memory [STM]) or 24 h (B, long-term memory [LTM]) later. (**D**) Discrimination index (DI) calculated during the STM (one-way ANOVA; *F*_2, 34_ = 6.936, *p* = 0.003) and LTM (*F*_2, 35_ = 7.749, *p* = 0.0016) testing. Data are reported ± the standard error of the mean. Asterisks indicate values significantly different (**p < 0.01; ***p < 0.001, unpaired *t*-tests in panels B and C, Fisher's LSD in panel D). ANOVA, analysis of variance; LSD, least significant difference; TLT, transcranial laser therapy.

Cognitive deficits are a consistent feature of blast-related behavioral effects.^25^ Figure 2 shows testing in NOR, which measures the normal tendency of rats to prefer a novel object (NO) to a previously presented familiar object (FO). During the NOR training session (Fig. 2A), all three groups (sham + sham TLT, blast + sham TLT, and blast + active TLT) spent similar time exploring the two objects. However, whereas the sham + sham TLT rats spent more timing exploring the NO than the FO in testing both 1 h (STM; *p* < 0.001, unpaired *t*-test) and 24 h (LTM; *p* < 0.01) later (Fig. 2B), blast-exposed rats that received sham TLT explored the two objects a similar amount of time. These deficits were not reversed by TLT treatment of blast-exposed animals. A discrimination index (DI), which calculates the relative tendency to explore the NO versus FO, also revealed that recognition memory deficits in blast-exposed rats were not rescued by TLT in either STM or LTM testing (Fig. 2C).

**FIG. 3.**
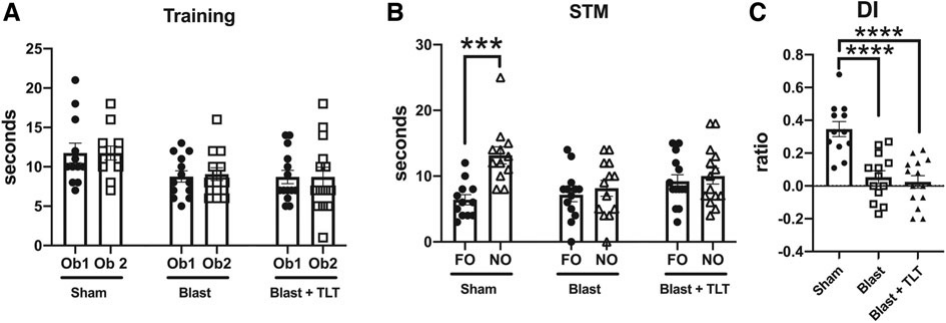
Novel object localization (NOL) testing of blast-exposed rats treated with TLT. Sham and blast-exposed rats were treated with sham TLT (sham and blast; *n* = 12 sham, *n* = 13 blast) or active TLT (blast + TLT; *n* = 14). Rats were tested in NOL at the end of treatment. (**A**) Time spent exploring the objects (Ob1 and Ob2) during the training session. (**B**) Exploration of the previously presented familiar object (FO) compared to the novel object (NO) when presented 1 h later (short-term memory; STM). (**C**) Discrimination index (DI) calculated during the STM testing (one-way ANOVA; *F*_2, 36_ = 18.12, *p* < 0.001). Data are reported ± standard error of the mean. Asterisks indicate values significantly different (***p < 0.001; ****p < 0.0001. unpaired *t*-tests in panel B, Fisher's LSD in panel C). ANOVA, analysis of variance; LSD, least significant difference; TLT, transcranial laser therapy.

A similar pattern was observed in NOL testing, which measures the tendency of normal rats to prefer exploring a FO in a novel location versus a familiar one (Fig. 3). All three groups explored the two objects equally during the training session (Fig. 3A). However, when tested 1 h later (STM; Fig. 3B), whereas the sham + sham TLT group spent more time exploring the FO moved to the novel location than the one in the same location (*p* < 0.001), blast + sham TLT exposed rats failed to spend more time exploring the FO in a novel location, a deficit that was not rescued by TLT treatment. Similar patterns were revealed when a DI was calculated (Fig. 3C). Thus, TLT therapy failed to rescue either altered recognition (Fig. 2) or spatial localization memory (Fig. 3) after blast exposure.

### No rescue of altered fear learning by transcranial laser therapy in blast-exposed rats

**FIG. 4.**
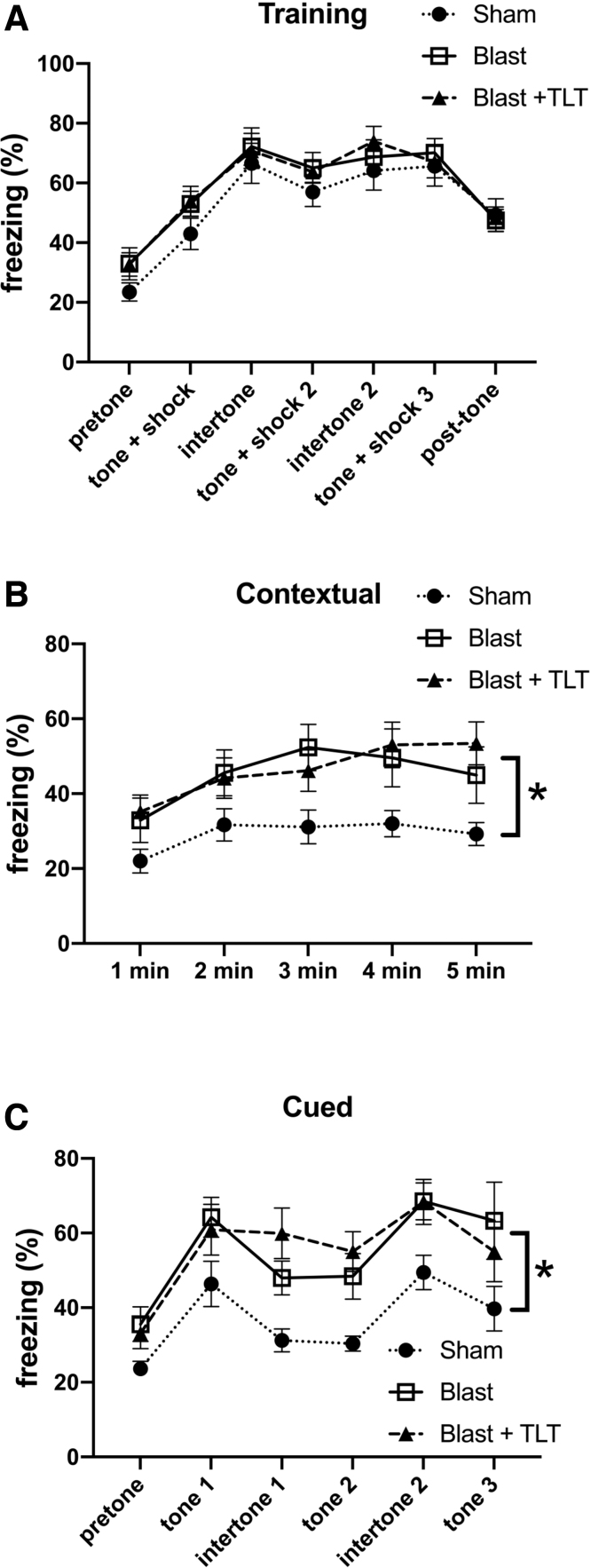
Fear learning in blast-exposed rats treated with TLT. Sham and blast-exposed rats were treated with sham TLT (sham and blast; *n* = 12 sham, *n* = 13 blast) or active TLT (blast + TLT; *n* = 14). Results are shown for the training phase (**A**), contextual fear memory (**B**), which was tested 24 h after training, and cued fear memory (**C**), which was tested another 24 h later. Pre-tone represents freezing before the first presentation of the tone/shock pairing. A repeated-measures ANOVA of freezing during the training sessions revealed a significant within-subjects effect of freezing across the training sessions for all groups combined (*F*_3.790, 136.449_ = 46.713, *p* < 0.001) without any significant interaction effect of freezing*condition (*F*_7.580, 136.449_ = 0.368, *p* = 0.93) and with no significant between subjects effects (*F*_2, 36_ = 1.585, *p* = 0.219). There were differences between groups in contextual testing (*F*_2, 35_ = 3.490, *p* = 0.041; Fisher's LSD: sham vs. blast, *p* = 0.034; sham vs. blast + TLT, *p* = 0.02; blast vs. blast + TLT, *p* = 0.846). In the cued phase testing, a repeated-measures ANOVA comparing freezing in the pre-tone to first tone across all groups revealed increased freezing (*F*_1, 33_ = 47.044, *p* < 0.001) without interaction effects (*F*_2, 33_ = 0.220; *p* = 0.804). However, there were significant between-subjects effects (*F*_2, 33_ = 3.625, *p* = 0.038; Fisher's LSD: sham vs. blast, *p* = 0.017; sham vs. blast + TLT, *p* = 0.039; blast vs. blast + TLT, *p* = 0.610). A repeated-measures ANOVA comparing freezing from the pre-tone through tone 3 revealed a significant within-subjects effect (*F*_2.461, 81.199_ = 7.058, *p* = 0.001) without group interaction effects (*F*_4.921, 81.199_ = 0.850. *p* = 0.517). However, there were significant between-subjects effects (*F*_2, 33_ = 6.141, *p* = 0.005; sham vs. blast, *p* = 0.007; sham vs. blast + TLT, *p* = 0.003; blast vs. blast + TLT, *p* = 0.830). Data are reported ± standard error of the mean. Asterisks (*) indicate values significantly different (*p* < 0.05) between groups. ANOVA, analysis of variance; LSD, least significant difference; TLT, transcranial laser therapy.

Blast-exposed rats show altered fear learning at ≥12 weeks after blast exposure.^25^ Figure 4 shows testing of contextual and cued memory testing in blast-exposed rats after sham or active TLT. During the training session, all three groups responded with increased freezing after the tone/shock pairings (Fig. 4A). In the contextual testing (Fig. 4B) when rats were returned to the training environment but without any tone or shock presentation, among rats that received sham TLT, the blast + sham TLT froze more than the sham + sham TLT (*p* = 0.034, Fisher's LSD). This effect was not reversed by TLT treatment of blast-exposed rats (*p* = 0.846, blast + sham TLT vs. blast + active TLT). Cued testing, which measures freezing when the tone is presented in a novel context without a shock, was tested 24 h later (Fig. 4C). In response to the tone, blast + sham TLT rats froze more than sham + sham TLT exposed rats (*p* = 0.017). This effect was not rescued by TLT treatment (*p* = 0.830, blast + sham TLT vs. blast + active TLT). Thus, TLT therapy did not rescue altered fear learning in blast-exposed rats.

### Transcranial laser therapy does not improve anxiety-related traits in blast-exposed rats

**FIG. 5.**
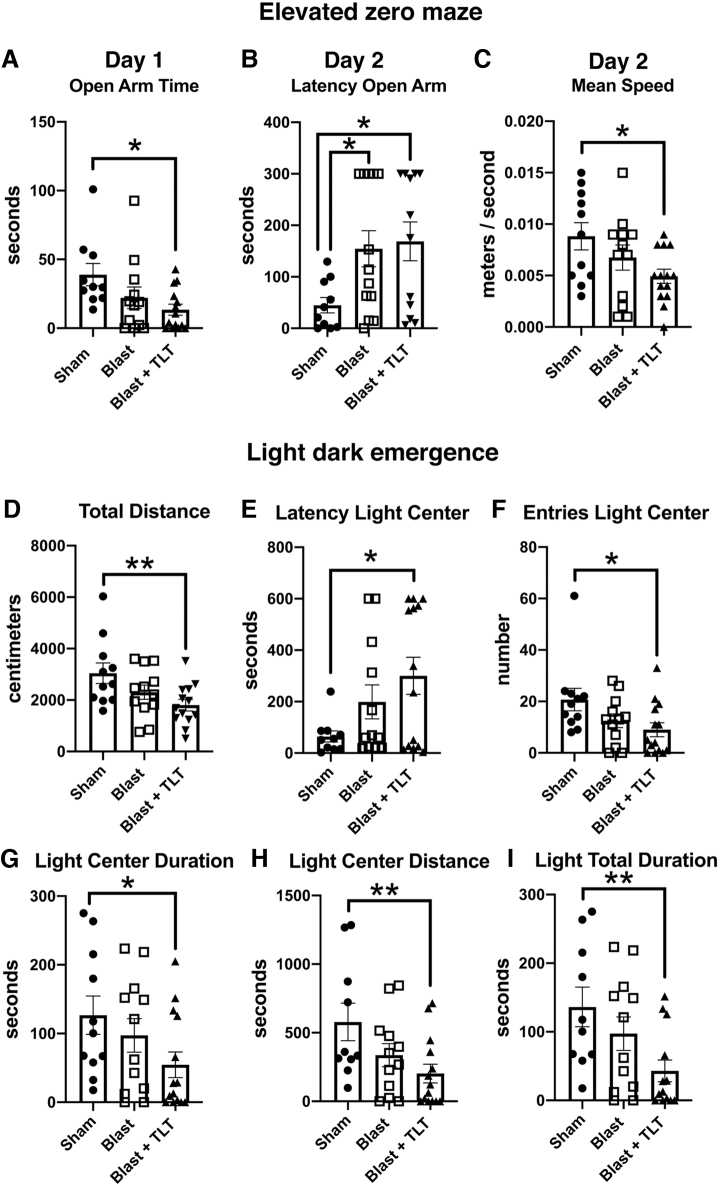
Testing of blast-exposed rats treated with TLT in elevated zero maze (EZM) and light dark (LD) emergence. Sham and blast-exposed rats were treated with sham TLT (sham and blast; *n* = 12 sham, *n* = 13 blast) or active TLT (blast + TLT; *n* = 14). Shown for EZM is open arm time on day 1 of testing (**A**; one-way ANOVA; *F*_2, 33_ = 3.717, *p* = 0.03). Latency to enter an open arm (**B**; *F*_2, 32_ = 3.931, *p* = 0.03) and mean speed (**C**; *F*_2, 34_ = 3.326, *p* = 0.047) are shown for day 2. For LD, emergence shown is distance moved (**D**; *F*_2, 33_ = 4.289, *p* = 0.02), latency to enter the light center (**E**; *F*_2, 33_ = 3.444, *p* = 0.04), number of light center entries (**F**; *F*_2, 34_ = 3.371, *p* = 0.046), time spent in the light center (**G**; *F*_2, 32_ = 4.216, *p* = 0.023), distance moved in the light center (**H**; *F*_2, 33_ = 3.942, *p* = 0.03), and time spent on the light side (**I**; *F*_2, 32_ = 4.216, *p* = 0.03). Error bars indicate the standard error of the mean. Asterisks indicate significant group differences (*p < 0.05; **p < 0.01, Fisher's LSD). ANOVA, analysis of variance; LSD, least significant difference; TLT, transcranial laser therapy.

Anxiety is a related core feature of human PTSD and is found in blast-exposed rats.^25^ Figure 5 shows testing for anxiety-related traits in an EZM and an LD task. On day 1 of EZM testing, the only difference between groups was that blast + TLT rats spent less time in the open arms than sham + sham TLT (*p* = 0.0104). On day 2, compared to sham + sham TLT, blast + active TLT rats moved slower (*p* = 0.014), and both blast + sham TLT (*p* = 0.026) and blast + active TLT (*p* = 0.014) exhibited longer latencies to enter an open arm. In LD emergence testing, compared to sham + sham TLT, blast + active TLT moved less (*p* = 0.0062), exhibited a longer latency to enter the light center (*p* = 0.013), made fewer light-center entries (*p* = 0.015), spent less time in the light center (*p* = 0.035), moved less in the light center (*p* = 0.0085), and spent less time on the light side (*p* = 0.0074), even though, in this task, blast + sham TLT did not significantly differ from sham + sham TLT. Thus, TLT did not improve anxiety-related features after blast exposure, and blast + active TLT rats exhibited features of anxiety that were not apparent when blast + sham TLT was compared to sham + sham TLT.

### Transcranial laser therapy does not affect blast-related pathological changes

**FIG. 6.**
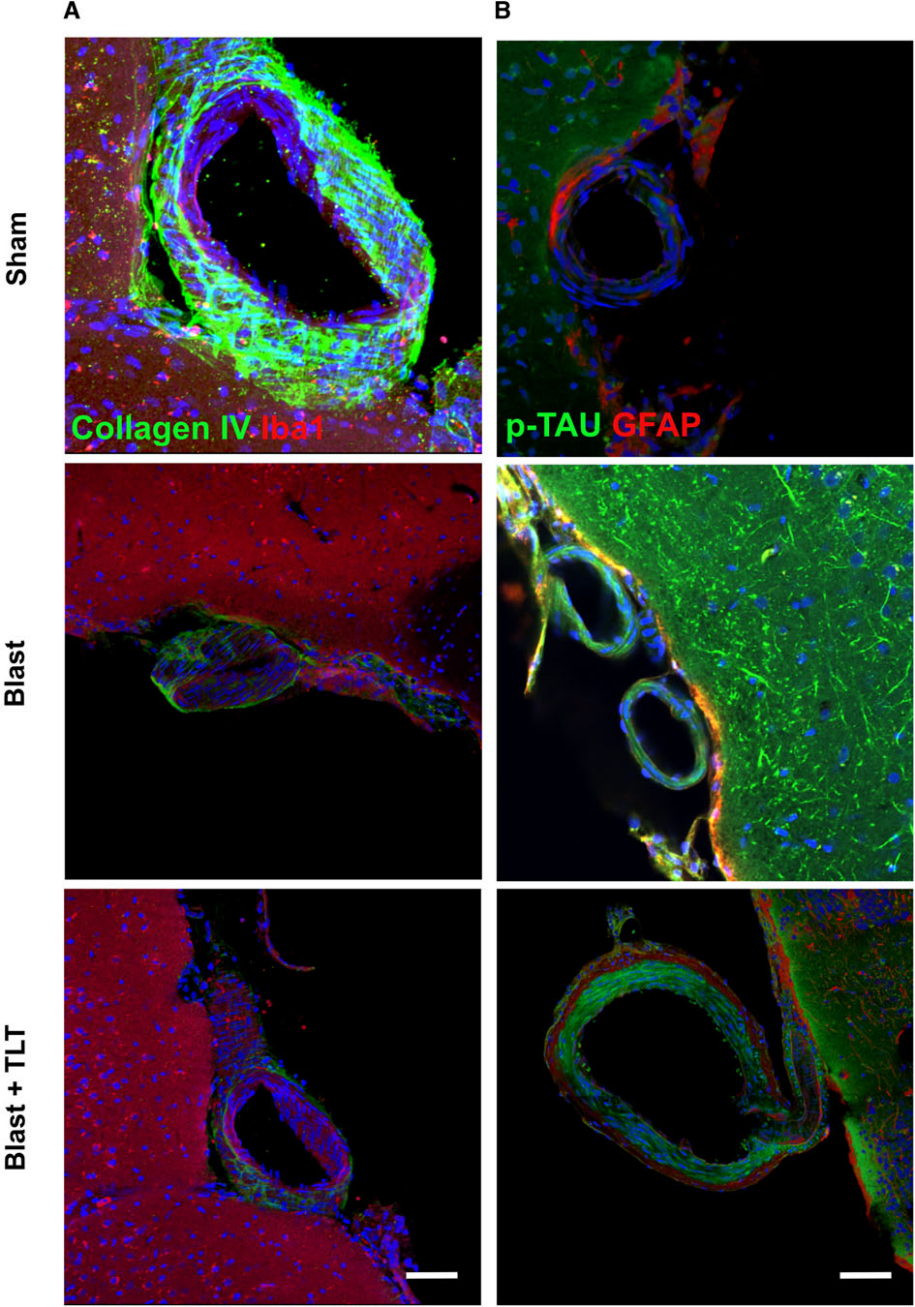
Chronic vascular pathology after blast exposure is not reversed with TLT. (**A**) Motor cortex from rats euthanized after TLT treatment. Sections were immunostained for collagen IV (green) and Iba1 (red). Blast-exposed rats treated with TLT or sham TLT show loss of collagen from the cortical vessel compared with non-blast-exposed rats treated with sham TLT (sham). (**B**) Brain sections were immunostained for p-tau (red) or GFAP (green). In rats exposed to sham TLT, note the increased p-tau staining in blast-exposed versus sham. This increased staining was not affected by TLT. Scale bars: 10 μm. GFAP, glial fibrillary acidic protein; Iba1, ionized calcium-binding adaptor molecule 1; TLT, transcranial laser therapy.

**FIG. 7.**
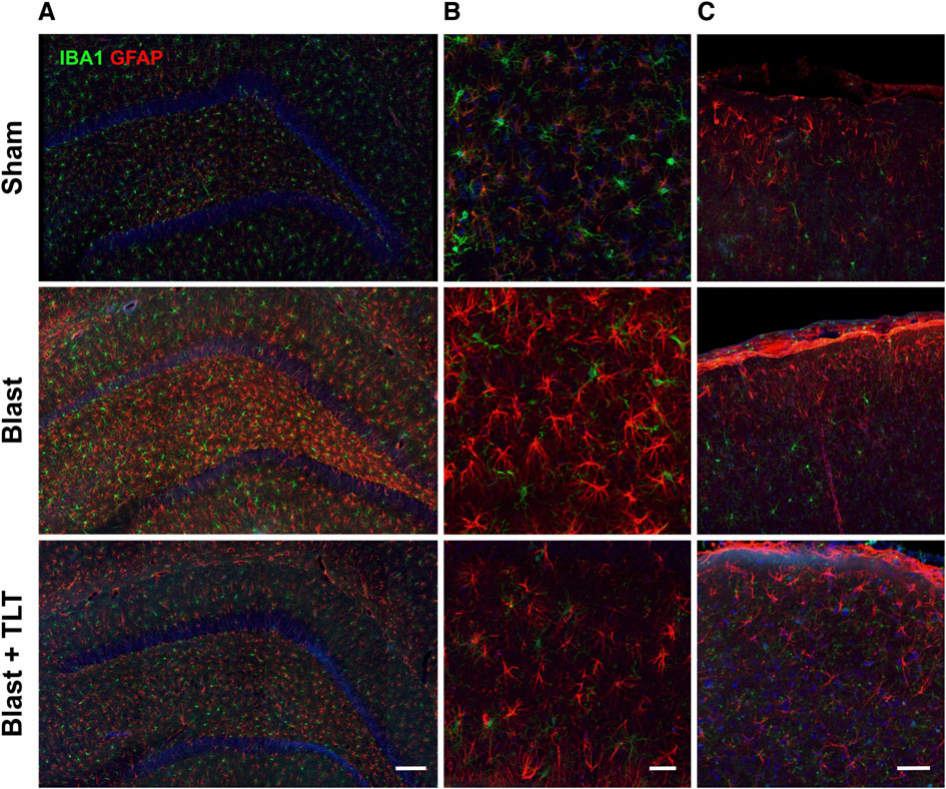
Absence of TLT effect on astrogliosis in blast-exposed rats. Sections through the hippocampus (**A**), hippocampal hilar region (**B**), or layer I of neocortex (**C**) immunostained for Iba1 (green) or GFAP (red). In rats exposed to sham TLT, there was increased GFAP staining in the blast-exposed compared to the sham. TLT treatment did not appear to appreciably affect this increased staining. There were no obvious differences in microglial staining (Iba1). Scale bar: 100 (A), 10 (B), and 20 μm (C). GFAP, glial fibrillary acidic protein; Iba1, ionized calcium-binding adaptor molecule 1; TLT, transcranial laser therapy.

Blast exposure induces a variety of pathological effects in this model, including vascular pathology and accumulation of abnormally phosphorylated tau.^7,37–39^ To determine whether TLT might improve any of these blast-related pathologies, we examined brain sections collected from animals euthanized at the end of behavioral testing. Figure 6 shows sections immunostained for collagen IV and Iba1. Blast-exposed rats treated with TLT or sham TLT showed loss of collagen IV from cortical vessels compared to non-blast-exposed rats treated with sham TLT. In sections immunostained for p-tau and GFAP, compared to sham + sham TLT, blast-exposed rats—whether or not they were treated with TLT—exhibited perivascular p-tau accumulations similar to those previously reported.^7^ TLT treatment also failed to affect astrogliosis in blast-exposed rats (Fig. 7).

## Discussion

Beneficial effects of TLT have been shown in a range of neuro- and psychological disorders in both humans and animal models.^13,17–20^ In mice, for example, laser treatments administered three times a week over a period of 4 weeks improved deficits in learning and memory induced by unpredictable chronic mild stress as well as reverted altered levels of brain nitric oxide, reactive oxygen species (superoxide dismutase), and serum cortisol levels.^40^ Another study in mice showed that 20 days of TLT had antianxiety and -depressive effects after chronic restraint stress.^41^ TLT has also been found to be effective in relieving depressive features induced by chronic mild stress in rats.^36^

Rats exposed to low-level repetitive BOP injuries develop a variety of cognitive and PTSD-related behavioral traits.^21–25,42^ To examine the effects of TLT treatment on these traits, we used a three-group study design, which included a non-blast-exposed group treated with sham TLT and two blast-exposed groups treated with sham or active TLT. In this design, comparison of blast + sham TLT to sham + sham TLT served as a positive control for the appearance of the blast-induced behavioral phenotype. Comparing blast + TLT to blast + sham TLT allowed the effectiveness of the TLT treatment to be determined. We found that TLT was unable to reverse blast-associated alterations in recognition memory, spatial memory, or fear learning and may have increased anxiety in blast-exposed rats. TLT treatment also failed to affect vascular pathology, perivascular p-tau accumulation, or astrogliosis in blast-exposed rats.

Multiple studies have reported beneficial effects of TLT in treatment of TBI in animal models.^26,29–33^ However, nearly all of these studies used models of non-blast-related TBI and addressed acute-to-subacute effects post-injury, unlike the studies here, which used a blast model of injury and addressed chronic effects present at >6 months after injury. Only one other study has addressed a role for TLT in a rat model of blast-induced neurotrauma.^33^ This study used a combination of TLT with optoacoustic ultrasound delivered 1 h after injury and found improvements in balance and working memory as well as upregulation of brain-derived neurotrophic factor mRNA and downregulation of proapoptotic caspase-3 in cortical neurons. This study thus differed from ours in its use of a combined treatment and focus on acute effects.^33^

Several limitations of our studies must be acknowledged, including the choice of wavelength and mode of operation. Various laser parameters have been used in TLT studies. Most studies in rodent models of non-blast TBI used wavelengths of 810 nm.^43,44^ Although there has been little systematic study of the effects of wavelength, one study in mice found that whereas 660 and 810 nm were effective, 732 and 980 nm were not.^45^ TLT can also be used in continuous wave and PW modes. Studies comparing the two modes in non-blast TBI have found the PW mode to be more effective.^26,29,45^ Thus, we chose to use an 810-nm, 10-Hz PW mode for the present studies. It, however, remains possible that an alternative wavelength or a continuous wave mode might be more effective for treating the chronic effects of blast injury.

The light emitted by TLT has been shown to have generally good penetrability of the skull, although it is subject to attenuation by factors including scalp and skull thickness, wavelength, light coherence, tissue thickness, and anatomical irradiation site.^43^ TLT administered at the bregma induces an extensive diffusion pattern in mice, rats, and rabbits that should illuminate the entire brain.^46^ Here, we administered TLT over the head in the midline on the dorsal surface of the rat head midway between bregma and lambda (Fig. 1). Placement between bregma and lamda was chosen to deliver the greatest irradiance to several central brain structures thought to be involved in the generation of PTSD-related behavioral traits, namely the amygdala, hippocampus, and anterior cortex.^24^ The choice of a laser probe/collimator held 0.05 mm above the skull also allowed a fairly broad area to be irradiated (4 mm; Fig. 1). We realize, however, that TLT applied more focally, directly to the skull, may result in deeper penetration and higher irradiation of specific brain structures.^44^ As more is known about this model, it may be of value to repeat these studies with a more targeted area of irradiation.

One particular factor to be considered in the present studies is the relative thickness of the rat skull compared to the mouse, which may limit irradiation of deeper structures including limbic structures. Arguing against this being a significant factor, two recent studies used comparable TLT irradiation parameters to investigate potential beneficial effects of early TLT for preventing PTSD-like traits from appearing in rats.^47,48^ In both studies, short courses of TLT treatment prevented later development of PTSD-related traits. However, these studies differed from ours in that the TLT treatment was administered immediately after the inciting psychological stress, and only short-term behavioral effects were studied.

A final limitation of these studies is the choice of the time period for treatment. In those studies that have investigated TLT in mice or rats post-TBI.^26,29–33^ TLT was applied immediately after injury for, at most, a few sessions. Thus, they provide little guidance for studies such as ours concerning chronic effects. Whereas some studies in acute TBI models have suggested that prolonged daily treatments with TLT may be deleterious,^45^ most studies in chronic behavioral models have used treatment regimens of several weeks.^35,36,40,41,49^ Among animal studies, seemingly the most relevant to the present work are studies in rats utilizing chronic mild stress^36^ or reserpine^35^ to induce depressive-like behavior. In these studies, TLT administered over weeks to months improved depressive features.^35,36^ In humans suffering from the chronic effects of TBI, TLT has been used for up to 5 years without apparent toxicity, with some case reports describing sustained and dramatic improvements.^27,28,50,51^

In the current studies, we applied TLT treatment for 5 days per week for 6 weeks, reasoning that this would be a sufficient treatment course to assess whether any beneficial effect was to be observed. The fact that, on some anxiety measures, blast + active TLT-treated rats exhibited features of anxiety that were not apparent in blast + sham TLT-treated rats suggests that TLT had an effect, but not the desired effect. However, it remains possible that longer or shorter courses might be more effective.

## Conclusion

TLT has exerted promising effects in many neuro- and psychological disorders in humans and animal models. Thus, future studies may still be warranted in blast injury. However, based on the findings presented here, TLT may not be a promising treatment for the chronic cognitive and mental health problems that follow blast injury, at least if applied in the late intervention window studied here.

## Abbreviations Used

ANOVA: analysis of variance
BOP: blast overpressure
CS: conditioned stimulus
DI: discrimination index
EZM: elevated zero maze
FO: familiar object
GaAIAs: gallium aluminum arsenide
GFAP: glial fibrillary acidic protein
Iba1: ionized calcium-binding adaptor molecule 1
IgG: immunoglobulin G
LD: light/dark emergence
LEDs: light-emitting diodes
LSD: least significant difference
LTM: long-term memory
NO: novel object
NOL: novel object localization
NOR: novel object recognition
PBS: phosphate-buffered saline
PTSD: post-traumatic stress disorder
PW: pulsed wave
STM: short-term memory
TBI: traumatic brain injury
TLT: transcranial laser therapy
US: unconditioned stimulus
WRAIR: Walter Reed Army Institute of Research
